# Laparoscopic treatment for celiac artery stenosis caused by median arcuate ligament compression with Adachi V type vascular anomaly: a case report

**DOI:** 10.1186/s40792-021-01226-3

**Published:** 2021-06-09

**Authors:** Hiroshi Saito, Koichiro Sawada, Jyunichi Ogawa, Masashi Hashimoto, Masahiro Oshima, Masahiro Hada, Yosuke Kato, Kaeko Oyama, Masanori Kotake, Takuo Hara

**Affiliations:** Department of Surgery, Koseiren Takaoka Hospital, Eirakutyou 5-10, Takaoka, Toyama Japan

**Keywords:** Median arcuate ligament compression, Median arcuate ligament dissection, Adachi vascular anomaly

## Abstract

**Background:**

Median arcuate ligament syndrome (MALS), which results from compression of the median arcuate ligament (MAL), is a rare cause of abdominal pain and weight loss. Treatment is dissection of the MAL; however, the laparoscopic procedure is not yet established and it involves the risk of major vascular injury, especially in cases with an anomaly.

**Case presentation:**

A 47-year-old man was evaluated at the hospital for epigastric pain. Contrast computed tomography scan revealed stenosis of the celiac artery origin due to the MAL. An Adachi V type vascular anomaly was also observed. Laparoscopic treatment was performed to release pressure on the celiac artery. Laparoscopic ultrasonography was used to less invasively confirm the release of the MAL. Despite a concomitant Adachi V type vascular anomaly, surgery was safely performed using the laparoscopic magnification view and intraoperative ultrasonography. Follow-up ultrasonography confirmed the celiac artery stenosis has not recurred.

**Conclusions:**

A rare case of MALS with an Adachi V type vascular anomaly is presented and the laparoscopic treatment is detailed.

## Background

Median arcuate ligament syndrome (MALS) is a rare disease that may cause postprandial abdominal pain, nausea and vomiting, and weight loss [1]. The median arcuate ligament (MAL) is a fibrous band that connects the left and right diaphragmatic crura across the aortic hiatus at the level of T12/L1 vertebral bodies. In most cases, it may be superior to the celiac artery [2]. Radical treatment for MALS involves ligament dissection. Although the laparoscopic approach for treatment of MALS has been recently reported [3, 4], the standard procedure for laparoscopic MAL dissection has not yet been established. This case report describes laparoscopic MAL dissection for a patient with MALS and an Adachi V type vascular anomaly.

## Case presentation

A 47-year-old man was evaluated at the department of gastroenterology because of abdominal pain. He had a 1-year history of occasional epigastric pain. Contrast computed tomography revealed celiac artery stenosis caused by compression of the MAL. The patient was referred to the authors’ hospital for treatment.

**Fig. 1:**
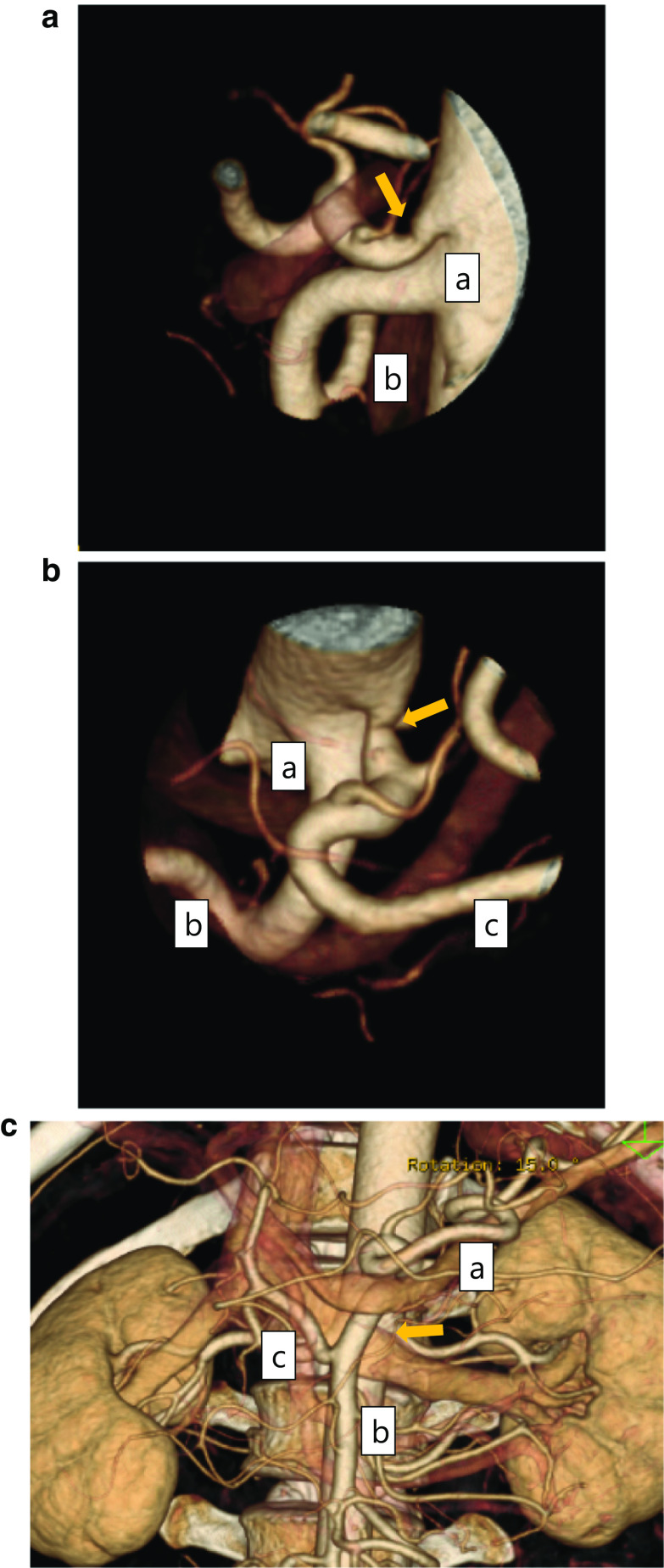
Preoperative abdominal computed tomography scan. **a**, **b** The origin of the celiac artery was stenosed due to compression of the median arcuate ligament (MAL): **a** superior mesenteric artery, **b** common hepatic artery, **c** splenic artery. **c** Adachi V vascular anomaly was detected: **a** splenic artery, **b** superior mesenteric artery, **c** common hepatic artery

Physical examination revealed epigastric tenderness. There was no bruit in the upper abdomen. The patient’s height was 167 cm weight was 69 kg. Laboratory findings and results of esophagogastroduodenoscopy imaging were normal. Detailed, contrast enhanced, three-dimensional computed tomography showed the origin of the celiac artery was narrowed due to compression of the MAL (Fig. 1a, b). In addition, an Adachi V type vascular anomaly was observed (Fig. 1a–c). The patient chose to undergo laparoscopic treatment.

**Fig. 2:**
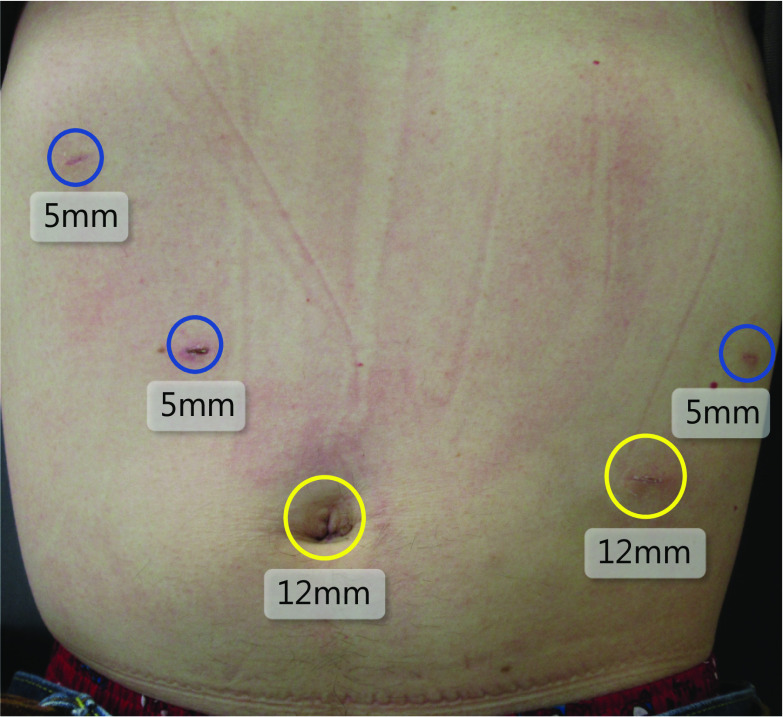
Abdominal surgical wound. The operation was performed with five ports

The procedure was performed under general anesthesia. The patient was placed in the supine position. A Stryker 1588 AIM camera system with 4K surgical display was used (Stryker, Kalamazoo, MI, USA). The first port of the scope was inserted via an open technique at the umbilicus. After that, four operating ports were inserted (Fig. 2). The left segment of the liver was elevated with a silicon disc (Hakko Co., Nagano, Japan). After dissecting the lesser omentum, the right crus of the diaphragm was identified, and the anterior surface of right crus was detached. The left gastric artery and vein were then exposed on the suprapancreatic surface, and the left gastric vein was resected.

**Fig. 3:**
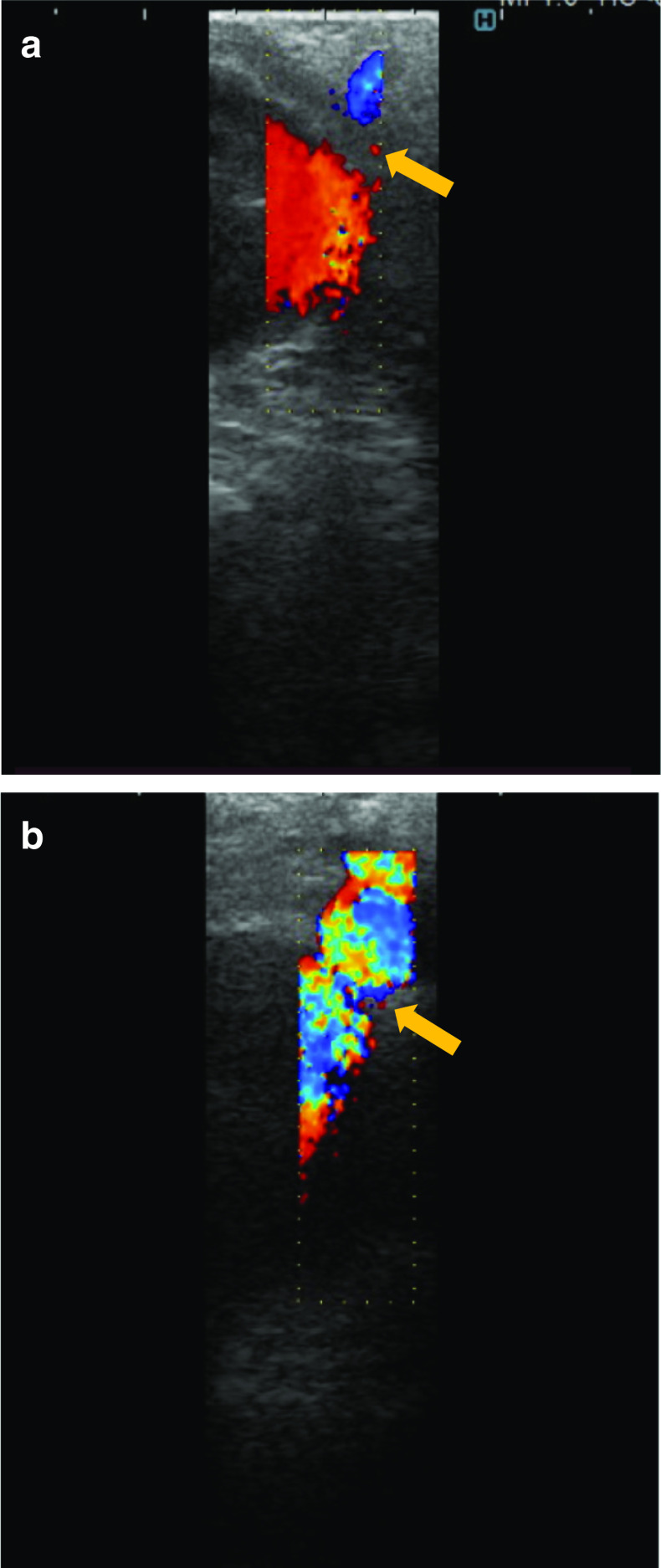
Laparoscopic ultrasonography. **a** Stenosis of the celiac artery origin was confirmed. **b** After MAL resection, release of the stenosis was confirmed

**Fig. 4:**
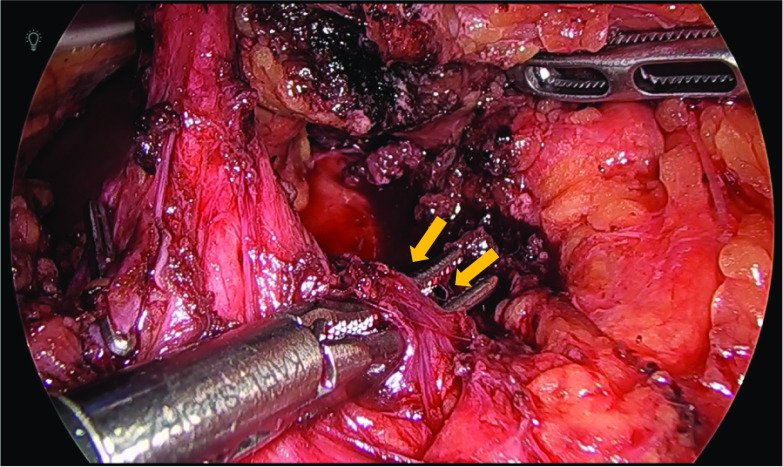
Laparoscopic findings. A fibrous connective tissue was found on the anterior aspect of celiac artery, which was confirmed as the MAL

An Adachi V type vascular anomaly was observed in which the common hepatic artery branched from the superior mesenteric artery. Stenosis of the celiac artery origin was confirmed by laparoscopic ultrasound (Fig. 3a). Fibrous connective tissue on the anterior aspect of celiac artery was observed, which was identified as the MAL (Fig. 4). After dissecting the MAL, the origin of the celiac artery was identified, and release of stenosis was confirmed by laparoscopic ultrasound (Fig. 3b).

**Fig. 5:**
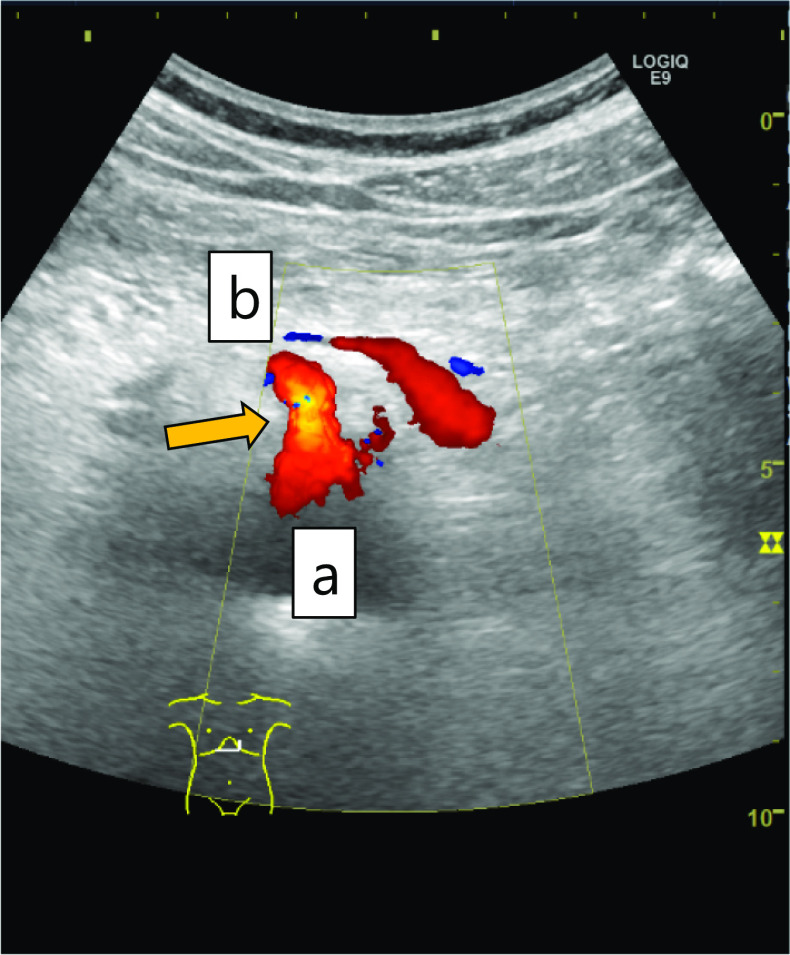
Postoperative ultrasonography. No stenosis was seen at the celiac artery origin: **a** aorta, **b** celiac artery

The patient’s postoperative course was uneventful. He was discharged on postoperative Day 5, and his symptoms of abdominal pain improved. At 2-month follow-up, ultrasonography was performed and showed no stenosis at the origin of the celiac artery (Fig. 5).

## Discussion

MALS is characterized as a celiac artery compression syndrome, and it causes postprandial epigastric pain, nausea and vomiting, and weight loss. This syndrome was first described in a case report by Harjola et al. in 1963 [5]. The mechanism responsible for the pain is not completely understood. One of the most accepted theories is that the compressed celiac trunk may cause limited blood flow and organ ischemia, which results in abdominal pain [6]. Second, MALS causes symptoms due to mesenteric ischemia as a consequence of a “steeling” syndrome. Blood flow from the superior mesenteric artery is diverted through the collateral circulation to compensate for the impaired blood flow through the stenosed celiac artery [7]. Third, the abdominal nerve plexus is adjacent to the MAL; therefore, abdominal pain can be caused by direct stimulation of the sympathetic nerves [6]. In this case, an Adachi V type vascular anomaly was detected. It is possible that MALS is related to the presence of a vascular anomaly, although previous reports did not address this potential relationship.

The treatment for MALS includes the surgical release of the celiac artery compression with dissection of the MAL. Endovascular angioplasty or stenting are not recommended because of the risk for recoil restenosis, dissection, and fracture of the stent [8]. However, chronic compression of the celiac artery may lead to luminal stenosis by intimal hyperplasia, medial fibrodysplasia, and disorganization of the adventitia. For these reasons, surgical treatment of the celiac artery compression does not always relieve symptoms. Previous reports noted intraluminal treatment like angioplasty or splanchnic revascularization may be needed as the additional treatment if symptom remission cannot be obtained after surgical treatment [7].

Recently, use of the laparoscopic approach for MAL treatment has been reported [3, 4]. The laparoscopic magnification view and wide working space make it possible to identify the anatomic orientation of the MAL. In this case, an Adachi V type vascular anomaly was found, which is very rare—the occurrence rate of this anomaly is 0.4% [9]. Extreme caution is needed while performing exposure of the vessels. Preoperative visualization using three-dimensional computed tomography and intraoperative identification of the vascular positional relationship are important. In this case, a highly accurate camera system and 4K monitor enabled the surgical team to achieve a good view of the MAL and to perform delicate manipulation during the surgery. This approach also includes assessment of celiac artery flow before and after dissection of the MAL by laparoscopic ultrasonography. This author’s team believes that ultrasound imaging is critical to use as an integral part of the surgery because it provides information regarding the degree of compression.

## Conclusions

This case report described a rare case of MALS with an Adachi V type vascular anomaly. With a laparoscopic procedure, safe release of the MAL was performed.

## Data Availability

This case report does not have a dataset. All related data are included within the article.

## Abbreviations

MALS: Median arcuate ligament syndrome
MAL: Median arcuate ligament

## References

[1] Dunbar JD, Molnar W, Beman FF (1965). Compression of the celiac trunk and abdominal angina. Am J Roentgenol Radium Ther Nucl Med.

[2] Manghat NE, Mitchell G, Hay SC (2008). The median arcuate ligament syndrome revisited by CT angiography and the use of ECG gating-a single centre case series and literature review. Br J Radiol.

[3] Okada H, Ehara K, Ro H (2019). Laparoscopic treatment in a patient with median arcuate ligament syndrome identified at the onset of superior mesenteric artery dissection: a case report. Surg Case Rep.

[4] Roayaie S, Jossart G, Gitlitz D (2000). Laparoscopic release of celiac artery compression syndrome facilitated by laparoscopic ultrasound scanning to confirm restoration of flow. J Vasc Surg.

[5] Harjola PT (1963). A rare obstruction of the coeliac artery; report of a case. Ann Chir Gynaecol Fenn.

[6] Sun Z, Zhang D, Xu G (2019). Laparoscopic treatment of median arcuate ligament syndrome. IRDR.

[7] Duffy AJ, Panait L, Eisenberg D (2009). Management of median arcuate ligament syndrome: a new paradigm. Ann Vasc Surg.

[8] Glen S (2009). Roseborough: laparoscopic management of celiac artery compression syndrome. J Vasc Surg.

[9] Adachi B, Hasebe K (1928). Das Arteriensystem der Japaner, Bd, II.

